# RF-Alphabet: Cross Domain Alphabet Recognition System Based on RFID Differential Threshold Similarity Calculation Model

**DOI:** 10.3390/s23020920

**Published:** 2023-01-13

**Authors:** Yajun Zhang, Yan Yang, Zijian Li, Zhixiong Yang, Xu Liu, Bo Yuan

**Affiliations:** 1 School of Software, Xinjiang University, Urumqi 830091, China; 2 College of Information Science and Engineering, Xinjiang University, Urumqi 830046, China

**Keywords:** gesture recognition, RFID, tags, signal processing

## Abstract

Gesture recognition can help people with a speech impairment to communicate and promote the development of Human-Computer Interaction (HCI) technology. With the development of wireless technology, passive gesture recognition based on RFID has become a research hotspot. In this paper, we propose a low-cost, non-invasive and scalable gesture recognition technology, and successfully implement the RF-alphabet, a gesture recognition system for complex, fine-grained, domain-independent 26 English letters; the RF-alphabet has three major advantages: first, this paper achieves complete capture of complex, fine-grained gesture data by designing a dual-tag, dual-antenna layout. Secondly, to overcome the disadvantages of the large training sets and long training times of traditional deep learning. We design and combine the Difference threshold similarity calculation prediction model to extract digital signal features to achieve real-time feature analysis of gesture signals. Finally, the RF alphabet solves the problem of confusing the signal characteristics of letters. Confused letters are distinguished by comparing the phase values of feature points. The RF-alphabet ends up with an average accuracy of 90.28% and 89.7% in different domains for new users and new environments, respectively, by performing feature analysis on similar signals. The real-time, robustness, and scalability of the RF-alphabet are proven.

## 1. Introduction

With the rapid development of gesture recognition technology and wireless technology, human gesture recognition has received increasing attention from academia and industry [1]. Researchers have adopted different approaches to achieve fine-grained human perception [2,3]. Human gesture perception technology is an emerging branch of human perception technology, and the applications involved in it have a significant impact on our daily life: e.g., virtual reality (VR), smart home, smart city, 5G communication, and sign language recognition technology [4,5,6,7,8,9]. Especially in the context of the current epidemic-ridden environment, RFID-based gesture recognition can be zero-contact, non-invasive, and can be applied to many places. For example, it can be used in hospitals, libraries, supermarkets, museums and other public places to avoid germ infection by gesture recognition of the human body. Unlike traditional keyboard input and voice input, gestures give users a better experience in noisy environments. RFID tags are everywhere and commonly used for bus cards, car keys, pass cards, etc. One main reason for the widespread usage is the simplicity and extremely low cost of the RFID tags (each tag costs 5–10 cents USD). With the development of 5G communication technology, it is possible to interact with specific smart devices by gesture input in daily family life.

Traditional solutions for gesture recognition are performed by users wearing specific wearable sensors or cameras [10,11,12,13,14,15,16,17]. Although these traditional solutions have high recognition rates, wearable sensor-based approaches tend to place an additional burden on the human body and wearing these devices outside the home is often accompanied by inconvenience and forgetfulness. The Kinect-based multi-level gesture recognition proposed by Feng et al. [18] uses component concurrent features and sequentially organized features to extract semantic units from frame segmentation and the entire gesture sequence features, and then classify motion, position and shape components sequentially. Thus, a relatively high single-learning gesture recognition performance is obtained. Although the accuracy of gesture recognition using camera-based gestures is high, it is sensitive to light and often does not provide high recognition in dark environments. It may even violate the user’s privacy [19].

In this paper, we explore a flexible, easy-to-deploy, non-intrusive gesture recognition mechanism. For wireless signals, when a user is performing specific gestures, these specific gesture transformations affect the RF signal (amplitude or phase) of the wireless channel. By analyzing these channel transformations, data processing and feature extraction, the recognition of gestures can be achieved. The literature [20] uses dynamic time warping (DTW) based pattern matching to achieve gesture classification. However, these traditional methods require an extensive data acquisition process and data preprocessing as well as a long time data training process. The performance of gesture recognition depends largely on the choice of feature extraction algorithm or the fit of the neural network.

**Figure 1:**
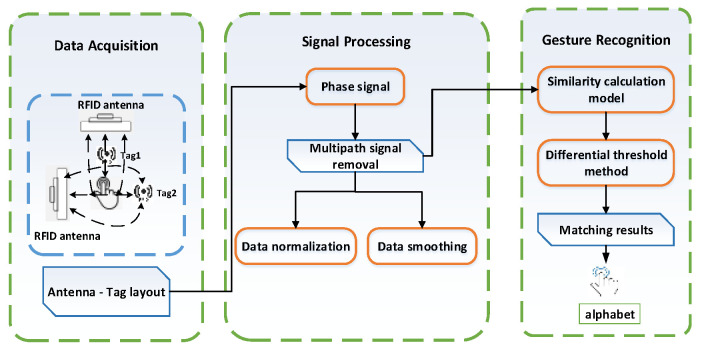
The system structure of RF-alphabet.

The model-based method has the advantages of fewer data sets, short training time and high scalability. Based on this idea, we designed a device-free, complex, fine-grained, domain-independent gesture recognition system, the RF-alphabet. As shown in Figure 1, a dual-antenna, dual-tag layout is used. Users draw 26 letters of the alphabet on the back of the tags. The RF-alphabet can map the captured signals to specific gestures. In the experiment, volunteers draw 26 letters in three different locations (dormitory, conference room, and classroom).

There are a number of problems and challenges encountered in designing the RF-alphabet system for traditional gesture recognition. First, in drawing the 26 gesture letters, the user dynamically draws the gesture letters behind the tag, which will design complexity, diversity, and fine-grained signal transformations. Current commercial RFID readers provide a limited spatial resolution of the signals (RSS and phase). How to capture these fine-grained transformations becomes one of the challenges. Second, gesture recognition designs complex spatiotemporal signal transformations. Among the 26 English letters, there exist some letters with very similar gesture signals. Often for some fine-grained gesture signals, RFID readers are not able to sensitively perceive their transformations. Moreover, in practical experimental sites, they are often accompanied by multipath effects [21], where the signals are received along with additional noise. It becomes one of the challenges to extract the fine-grained packing signal for gesture recognition. Finally, when a user is in one environment, its gesture recognition accuracy may be high, but when a different environment and a different user performs the gesture, the gesture recognition accuracy tends to drop sharply [22]. How to solve domain-independent gesture recognition becomes one of the challenges, and the properties of RF signals also increase the complexity of domain-specific feature extraction.

Given the above problems and the limited RF information provided by current commercial RFID readers, we look for solutions in its original signal and model design. As shown in Figure 1, in our experiments, we overcome the drawbacks of traditional multi-tag arrays, which are often accompanied by coupling effects. We use a specific antenna-tag layout with two antennas and two tags to successfully capture complex and fine-grained gesture signals. For gesture signals involving complex spatiotemporal transformations and multipath noise, we successfully achieve RF signal sensing capability after performing data smoothing, subtraction operations, data normalization, and data expansion on the raw data. We convert the original phase data, which seems to be irregular, into a 100 × 100 pixel picture. By designing and combining the models, we successfully converted the complex RF signals into recognizable waveband signals. Eventually, higher-level phase features were extracted to achieve feature extraction of temporal and spatial modes, which in turn accurately realized the gesture recognition of 26 English letters.

In the experiment, we conducted a large number of experiments in three different scenarios (dormitory, conference room, and classroom) and invited three volunteers (two men and one woman) to collect a sample set of about 8000 to evaluate the model.

The contributions of this paper are as follows: (1) RF-alphabet is a device-free, dual-tag-dual-antenna layout domain-independent 26 letters gesture recognition system. It overcomes the drawbacks of traditional gesture recognition using multi-tag arrays. (2) The RF-alphabet adopts a model-based design approach, which successfully achieves a low-cost, easy-to-deploy, and short training time model design. It overcomes the drawbacks of using traditional deep learning methods that require large data sets, long training time, and long experimental sample set collection time. (3) The RF-alphabet achieves cross-domain 26 letters gesture recognition on commercial RFID readers, which still has high accuracy for different domains due to the specific model design. After extensive experiments, The RF-alphabet shows that it has a flexible, easy-to-deploy, and highly scalable gesture recognition system. The average accuracy rate for the 26 letters was 92.43%.

The rest of the paper is organized as follows: Section 2 presents an overview of the research, Section 3 describes the preliminary work, Section 4 details the design of the 26-letter gesture recognition system, Section 5 discusses the implementation and evaluation of the experimental approach, Section 6 describes the research experiments and future research perspectives, and Section 7 summarizes the future research and the research summary.

## 2. Related Work

Current gesture recognition is divided into three main categories: gesture recognition based on wearable sensors, gesture recognition based on computer vision, and gesture recognition based on wireless technology. Among them, wearable-based gesture recognition technologies use wearable or nested sensing devices to capture hand or finger transformations. For example, inertial sensors are used to recognize eating gestures.

Gesture transformation of the signal [23]. A glove designed using a combination of accelerometer and gyroscope technology is used to track the gesture signals of seat transitions by wearing the glove. Although the adoption of wearable sensing devices is common in real life, people often tend to forget to wear related devices when they go out or put an extra burden on their bodies when wearing these devices.

Computer vision-based gesture recognition and human recognition systems use cameras or optical sensors to recognize gesture movements or human actions. In the context of the current deep learning environment, a large number of researchers have used gesture data trained by neural networks with high accuracy for testing specific actions performed by the human body. In the literature [24], Kinect is used to capture user gesture information and the captured data are used as pre-processed data for neural networks for feature training, which in turn are used to perform gesture recognition. In [21], an RGB camera is used for user gesture recognition. Although the gesture recognition obtained by computer vision-based methods has a high correct rate, these systems tend to be photosensitive and susceptible to lighting conditions. There is also the possibility of violating the user’s privacy. The non-intrusive nature of RF-alphabet is able to solve the drawbacks of the above methods well.

Gesture recognition based on wireless technology has received increasing attention from a large number of researchers because wireless devices have the advantages of being non-intrusive, easy to deploy, and highly scalable. Other wireless technologies such as WIFI [25], RFID, ultrasonic, and radar have been widely used for gesture recognition [22]. Yang et al. proposed the BVP (body-coordinate velocity profile) model for feature capture in independent domains to make full use of the channel information of WiFi for human activity sensing [26]. Wang et al. used acoustic signals to accurately identify gesture signals in the millimeter range and recognize them [27]. FingerPass uses channel state information (CSI) in WiFi to orderly authenticate users with finger gestures in smart homes, achieving high accuracy and low response latency. The real-time system involved in [28] Zhang et al.’s SMARS model using a commercial WiFi chipset is able to detect user sleep and assess sleep quality. Ubiquitous, non-intrusive and non-contact daily sleep detection was achieved. The RF-alphabet is able to sense complex and fine-grained alphabetic gesture information through a unique dual-antenna dual-tag layout and extract digital signal features through a Euclidean distance similarity prediction model, avoiding the disadvantages of traditional gesture recognition that requires a large number of experimental data sets and realizing high-precision and real-time gesture recognition. The RF-alphabet we designed also absorbs the advantages of wireless technology and applies it to human gesture recognition.

## 3. Preliminary

In this section, we introduce the RFID principle while considering the antenna layout and constructing the dual antenna-tag layout used in our experiments.

### 3.1. RFID Principle

A typical RFID system consists of a transmitter, receiver, microprocessor, antenna, and tag. The transmitter, receiver and microprocessor are generally combined together to become a reader; in RFID technology in the studio, the reader sends a signal, and after the connection of the antenna, the tag receives the signal and feeds back internal load information, and then back to the reader via the antenna, the reader identifies the information and then transmits the results to the host computer running in the background. The signal received by the reader, the backscattered signal S(t), which can be expressed as [29]:

(1) $$S\left(t\right)=a\left(t\right)e^{-j\theta \left(t\right)}=a(t)e^{-j(\theta_{0}+\frac{4\pi d}{\lambda })}$$

where a(t) and θ(t) are the amplitude and phase of the backscattered signal, respectively. j is an imaginary unit, and d is the distance between the antenna and the tag. Since the tag receives the signal from the antenna and generates a backscattered signal, the actual propagation distance should be 2d. $\theta_{0}$ is the initial offset caused by the device and contains the phase shift caused by the reader, the tag and the antenna. λ is the wavelength of the RF signal.

When the user crosses the detection area, the signal will propagate along three directions, including the direct path, the reflection path of the obstacle and the dynamic reflection path when the user moves. The backscattered signal S(t) is the superposition of dynamic and static reflection signals, the static reflection signal is the composite signal consisting of the direct path and the reflection path of the obstacle, and the dynamic reflection signal is the reflection signal when the user moves. When the user moves between the antenna-tag, assuming a total of n reflective paths coming from the user, then the received signal S(t) can be expressed as:

(2) $$S\left(t\right)=S_{s}\left(t\right)+S_{d}(t)$$

(3) $$S_{d}\left(t\right)=\sum_{n}a_{n}e^{-j(\frac{2\pi }{\lambda }\int d_{n}\left(t\right)dt+\mu )mod2\pi }$$

(4) $$S_{s}\left(t\right)=a_{s}e^{-j\theta_{s}}$$

where $S_{s}(t)$ is the static reflection signal, $S_{d}(t)$ is the dynamic reflection signal when the user is moving. $a_{n}$ is the amplitude of the user at the *n*th path, and $d_{n}\left(t\right)$ is the propagation distance at time t under path n.

We use phase information as the features of different gestures performed by the user. The gesture drawing of letters in the alphabet is fine-grained, and the traditional RSS information is not able to effectively distinguish the fine-grained gesture information, so we use phase information to extract the fine-grained gesture features.

### 3.2. Single Antenna-Tag Layout

**Figure 2:**
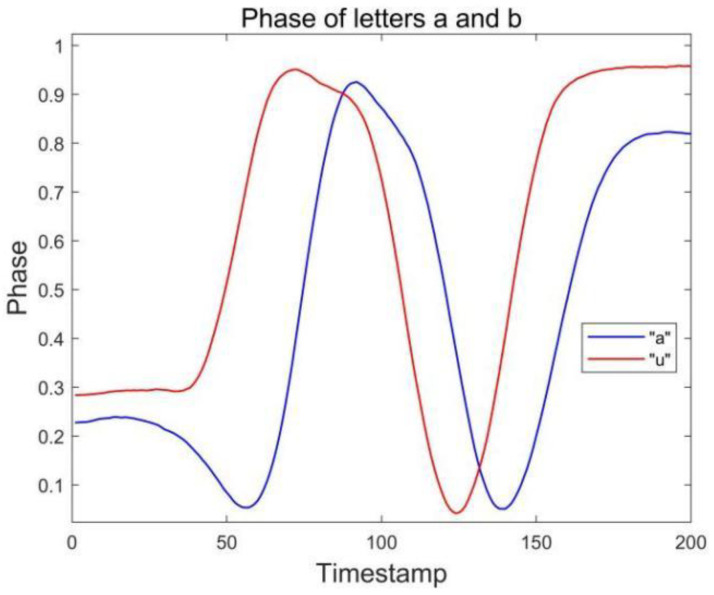
Phase of letters a and u collected by antenna #1.

Because there are similar waveforms (a and u, n and h) in the letters of the English alphabet, it is often difficult to distinguish the gesture signal waveforms when using the single antenna-single tag layout, which leads to recognition errors. At the beginning of the experiment, the pre-experiment is carried out by means of a single tag and single antenna combination. The results are shown in Figure 2, the phase waveforms of these similar letters are relatively similar, and there is great difficulty in recognition. Because it is necessary to analyze and recognize all the letters of the alphabet, if the single antenna and single tag layout are used for data acquisition, it will be difficult to distinguish similar letters when the number of recognized letters increases.

### 3.3. Dual Antenna-Dual Tag Layout

**Figure 3:**
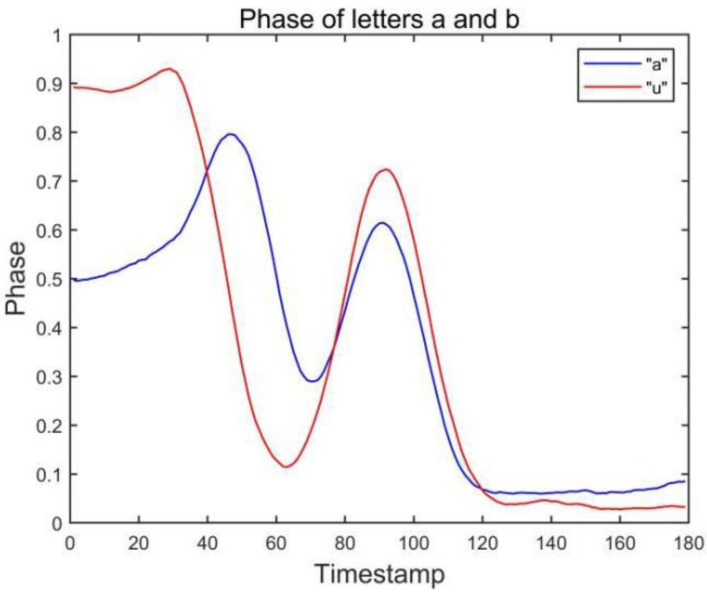
Phase of letters a and u collected by antenna #2.

It is difficult to distinguish similar letters in the layout of a single antenna and single label. Through our observation, we find that the layout of a double antenna and double label can effectively capture the drawn gestures. The user’s gesture is tracked in all directions by the antennas in front and to the right of the user. At the same time, the gesture features of the two antenna-tag arrays are fused to recognize the letter. Figure 3 shows the waveform captured by the side antenna. For similar letter pairs a and u, it can be found that when the waveform of the front antenna array is similar, the waveform captured by the side antenna array can effectively distinguish the two letters. It also further verifies the feasibility of the dual antenna-dual tag array.

### 3.4. Confused Letter Recognition

**Figure 4:**
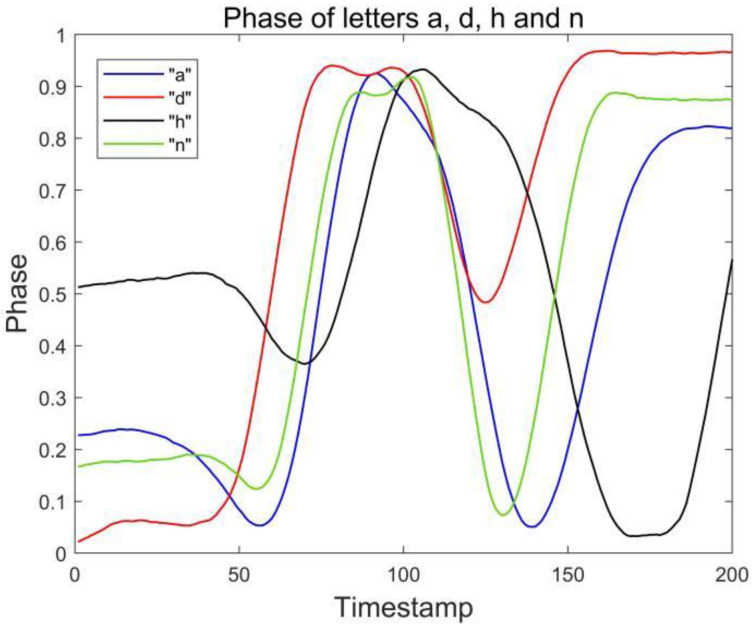
Phases of letters a, d, h and n collected by antenna #1.

**Figure 5:**
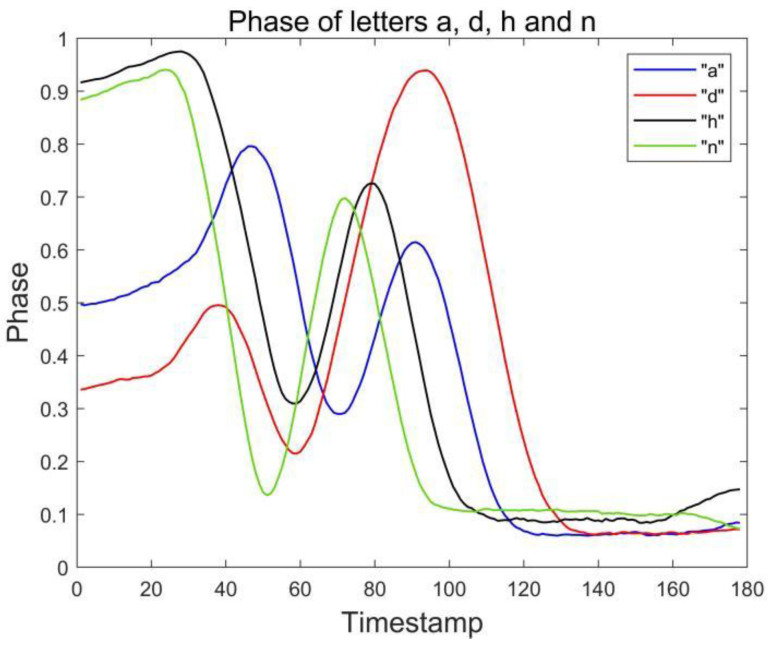
Phases of letters a, d, h and n collected by antenna #2.

During the experiment, we found that even through the combination of double tags and double antennas, we can largely solve the problem of similar signal waveforms of similar letters. However, there are still two groups of English letters that are difficult to distinguish, namely a and d, h and n, which are called confused letters. Figure 4 and Figure 5 show the phase waveforms of these two groups of letters after data preprocessing and normalization, respectively. Since the drawing gestures of each group of letters in the two groups are very similar, the phase waveforms collected by the two antennas are less different. For this case, the distinction can be made by comparing the feature point phase magnitude of the confused letters.

## 4. System Design

### 4.1. Signal Pre-Processing Module

Since the collected raw Phase is often accompanied by noise in the environment and influenced by different antennas and tags, it is not suitable to import the raw signal into the model as training data, so we first import the signal into the pre-processing module to improve the recognition ability of the signal.

1. Noise removal

The experimental deployment link is composed of two 9 dBi UHF gain antennas as well as two identical RFID UHF electronic tags. In the experiment the placement of the antennas and the distance between the tags often cause different effects; these effects include noise in the environment, the impact of the antennas themselves and the multipath effect between the tag and the tag, and between the tag and the antenna. In order to solve these problems we carried out the subsequent exploration. For the multipath effect, we cannot eliminate it, so we make appropriate adjustments to the experimental deployment by increasing the distance between tags and tags and tags and antennas, as well as trying different placement positions, as detailed in the experimental section, while ensuring the reception of the gesture signal to minimize its impact.

2. Data normalization

In RFID systems, due to the nature of tags and the different locations of human gesture movements, the collected raw phases may have different units of magnitude, which can make the comparability between data lower, thus reducing the accuracy of gesture recognition. The data normalization operation is to normalize the original data so that they are in the same order of magnitude to solve the comparability between data metrics, and at the same time, it can improve the convergence rate of the model as well as the accuracy of the model. Specifically, we use the min-max normalization method to linearly transform the output of the original data $X_{1}$:

(5) $$X_{1}=\frac{X-min}{max-min}$$

where *X* is the data after the subtraction operation, max is the maximum value of the current sample data, and min is the minimum value of the current sample data.

3. Data smoothing

**Figure 6:**
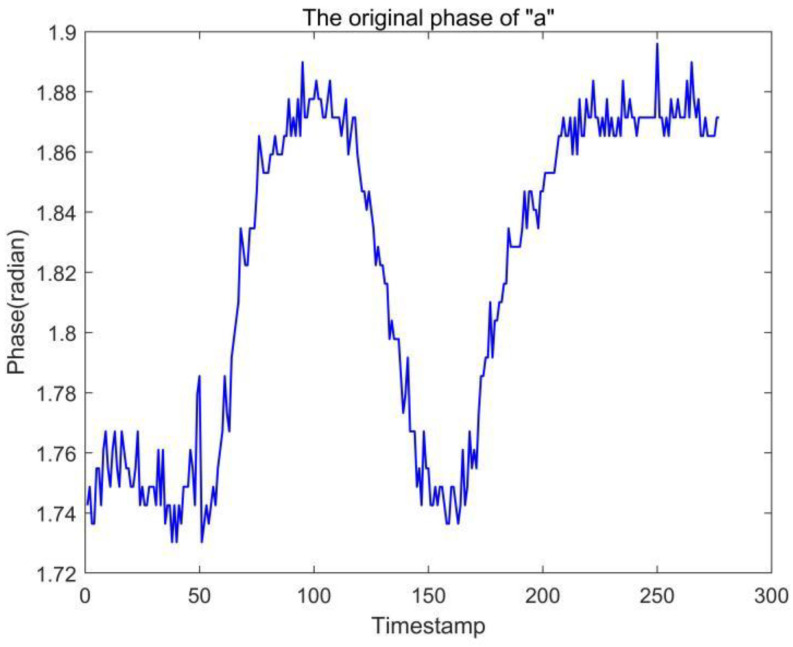
Label 1 outputs the raw phase information of letter a.

In gesture recognition experiments, the problem of excessive noise in the raw data is often encountered, such as the raw data “a” output from Tag1 shown in Figure 6. We can observe that the output “a” is too jittery, which makes it difficult to distinguish accurately in some cases, so we performed a simple smoothing process on the data.

In this paper, we use the Savitzky–Golay Filter to smooth the data because the Savitzky–Golay Filter is more suitable in RFID where data variation is dominant. Specifically, we set the width of the filter window to m=2n+1, the prediction point to $x=\left(t-n\right),t-1\ldots t,t+1,t+n)$, and fit the data within the window using a polynomial of k−1, and the fitting equation is shown in (6).

(6) $$y=a_{0}+a_{1}x+a_{2}x^{2}+\cdots +a_{k-1}x^{k-1}$$

For the system of equations as above to have a solution, then 2n+1≥k, where we take 2n+1≥k. Determine the fitting parameter *A* by least squares, which gives (7).

(7) $$\left(\begin{array}{c} y_{t-n}\\ \vdots \\ y_{t-1}\\ y_{t}\\ y_{t+1}\\ \vdots \\ y_{t+n} \end{array}\right)=\left(\begin{array}{ccccc} 1 & t-n & {\left(t-n\right)}^{2} & \cdots & {\left(t-n\right)}^{k-1}\\ \vdots & \vdots & \vdots & \vdots & \vdots \\ 1 & t-1 & {\left(t-1\right)}^{2} & \cdots & {\left(t-1\right)}^{k-1}\\ 1 & t & t^{2} & \cdots & t^{k-1}\\ 1 & t+1 & {\left(t+1\right)}^{2} & \cdots & {\left(t+1\right)}^{k-1}\\ \vdots & \vdots & \vdots & \vdots & \vdots \\ 1 & t+n & {\left(t+n\right)}^{2} & \cdots & {\left(t+n\right)}^{k-1} \end{array}\right)\left(\begin{array}{c} a_{0}\\ a_{1}\\ a_{2}\\ \vdots \\ a_{k-1} \end{array}\right)+\left(\begin{array}{c} e_{t-n}\\ \vdots \\ e_{t-1}\\ e_{t}\\ e_{t+1}\\ \vdots \\ e_{t+n} \end{array}\right)$$

We simplify the above matrix to obtain (8).

(8) $$Y_{(2n+1)\times 1}=X_{(2n+1)\times k}\times A_{k\times 1}+E_{(2n+1)\times 1}$$

The subscripts in the above equation indicate the respective dimensions, e.g., $A_{k\times 1}$ denotes a parameter with *k* rows and 1 column. We can find this by the least squares method $A_{k\times 1}$. The solution of

(9) $$A={\left(X^{T}\times X\right)}^{-1}\times X^{T}\times Y$$

The superscript *T* above denotes transpose. Then the predicted or filtered value of model Y is

(10) $$P=X\times A=X\times {\left(X^{T}\times X\right)}^{-1}\times X^{T}\times Y=B\times Y$$

Finally, we can obtain the relationship matrix between the filtered and observed values.

(11) $$B=X\times {\left(X^{T}\times X\right)}^{-1}\times X^{T}$$

In our experiments, we set the window length to 15 and the order to 3. After deriving the matrix B, we can quickly convert the observations to filtered values, thus achieving data smoothing.

### 4.2. Gesture Detection

The vast majority of the gesture phase signals between the 26 different letters have significant differences, and the few gestures with greater similarity can be narrowed down by the dual antennae with dual tags set up by the experiment. Therefore, we use the Phase signal as the only criterion for gesture detection. After obtaining the pre-processed signals, we embed them into the gesture detection module to further differentiate the recognition. The gesture detection module is divided into the following two parts: differential threshold estimation, and Dtsc model (similarity calculation + classification).

*A.*  Differential threshold method

The differential thresholding method is usually used as a fast algorithm for ECG QRS wave detection, which is essentially an algorithm for processing the signal. The main principle is that the rising slope or the falling slope of the ECG waveform is significantly different compared to other waveforms, so the location of the R-wave can be detected by detecting the derivative of the ECG signal sequence with respect to the time. In our experiments, we use the same method as in [30] to process the Phase signal and take the first-order derivative over zero and the second-order derivative extreme point as our R-wave position (i.e., eigenpoint), and then determine our threshold by the second-order difference Δy.

(12) $$\Delta y=y_{x+2}-{2y}_{x+1}+y_{x}$$

where, $y_{x}$ indicates the corresponding Phase value at the x timestamp.

*B.*  Dtsc model (Difference threshold similarity calculation).

For different alphabetic gesture information, the signal waveforms are often different, and the absolute differences in individual numerical characteristics can be reflected by Euclidean distance. For model selection, we use a combination of similarity calculation and threshold classification. From the above differential thresholding method we obtain the feature point set used to represent each letter $\left(x_{1},y_{1}\right),\left(x_{2},y_{2}\right),\left(x_{3},y_{3}\right),\left(x_{4},y_{5}\right)\ldots$, the mean value is obtained for each letter of the feature point set, and the first eight points are selected as the training feature point set due to the different lengths of the feature point sets obtained for different letters.

**Figure 7:**
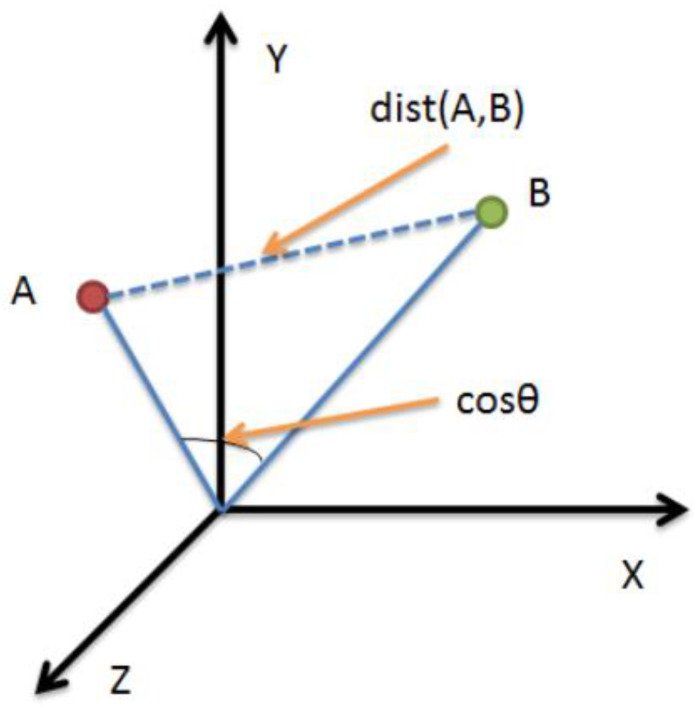
Calculating the distance between two points in n-dimensional space.

**Similarity calculation.** For the similarity algorithm selection, we used Euclidean distance to measure the similarity of different volunteers under the same gesture. Although Pearson’s correlation coefficient can distinguish independent and continuous phases, and small differences between different figures can be derived from the correlation coefficient, Euclidean distance provides great convenience for our subsequent classification problem. As shown in Figure 7, the similarity between two different gestures is calculated using the Euclidean formula, where dist(A,B) is tabulated as the distance between two points in three-dimensional space. Let, ${(X}_{i},Y_{i})$ be the different characteristic points of a volunteer under the same letter, and we Euclideanize it to yield

(13) $$\rho =\sum_{i=1}^{8}\sqrt{{\left(X_{i}-x_{i}\right)}^{2}+{\left(Y_{i}-y_{i}\right)}^{2}}$$

The smaller the equation above ρ, the greater the correlation between the letter plotted by the volunteer and the original letter.

**Threshold classification.** For the classification problem, we use the threshold values derived from the difference thresholding method as our classification criteria. Firstly, all the values derived from the recognition degree calculation are sorted, and then the threshold values are compared with the set of feature points of all letters for similarity calculation, and “n” (number of samples for each letter) data are selected in turn by comparison to achieve the function of gesture detection and recognition.

## 5. Implementation

**Figure 8:**
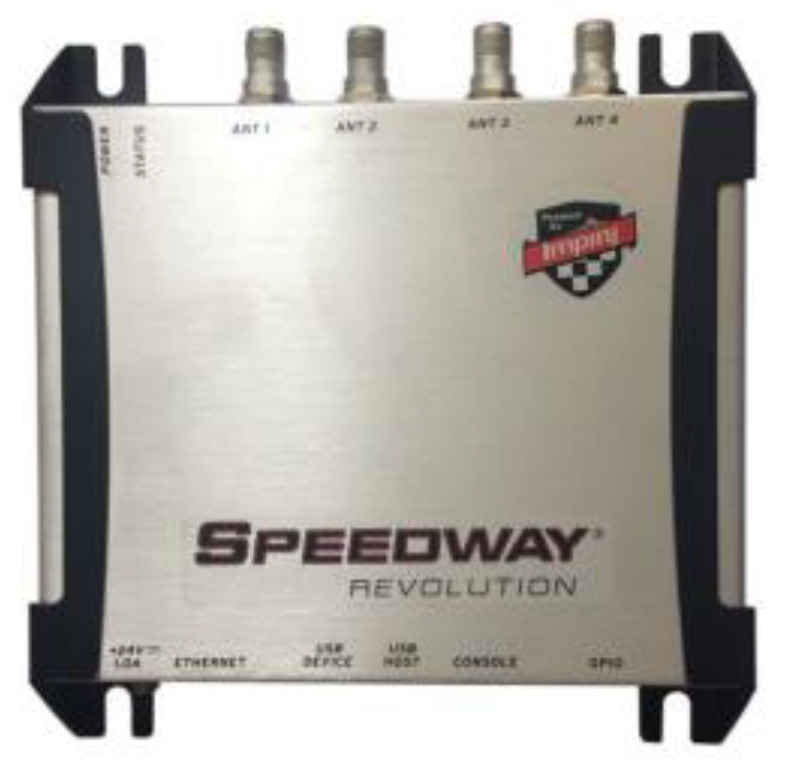
Impinj R420RFID Reader.

**Figure 9:**
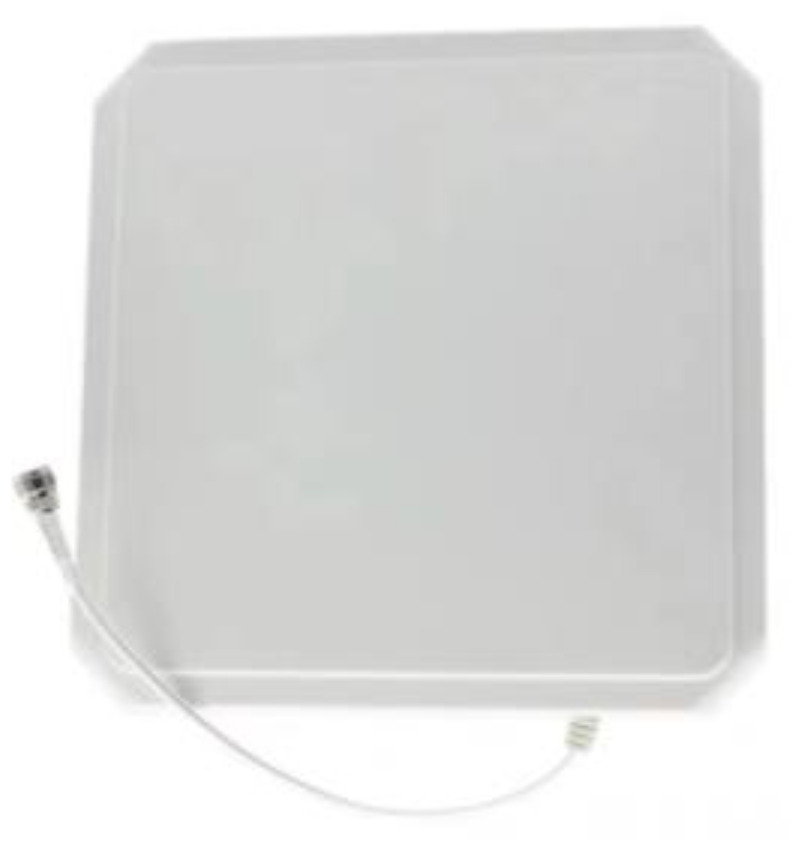
UHF circularly polarized antenna.

**Figure 10:**
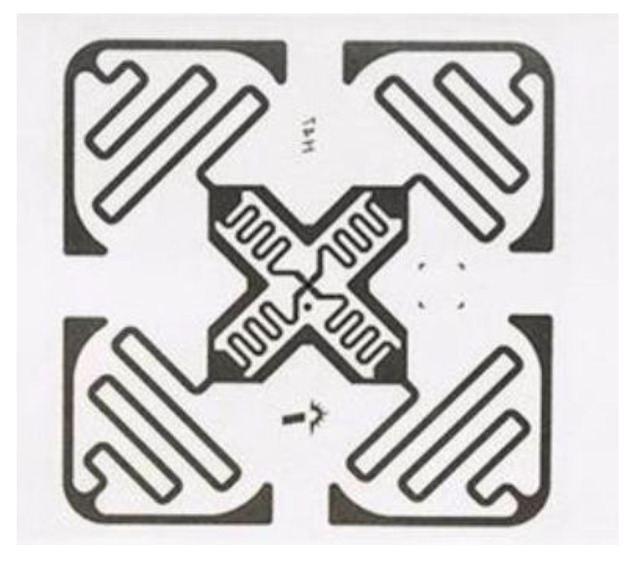
H47UHF Tag.

**Hardware:** The experimental equipment used is commercially available and does not require any modification. We use the H47UHF Tag in our experiments because of its high sensitivity, strong anti-interference ability, stable reading and writing ability and cheap price, a mere 5–10 cents. The experimental parameters are shown in Table 1. The hardware consists of four parts: an Impinj R420 RFID reader (Seattle, WA, USA; shown in Figure 8) operating at 920.875 MHz, two RFID UHF circularly polarized antennas (9 dBi) (shown in Figure 9), two 4 cm × 4 cm H47UHF tags (shown in Figure 10), and a Lenovo R7000p computer (Quarry Bay, Hong Kong).

**Software facilities:** we run our model on a Lenovo laptop equipped with a 2.5 GHz AMDR7 and 16 G memory for data acquisition and pre-processing. The RFID reader is connected to the laptop via an Ethernet cable and the neural model we have designed is implemented using Python.

**Data:** We invited eight volunteers (six males and two females) to collect a sample set of approximately 3500 samples, of which 2340 samples were used to fit out our data model and the rest were used to evaluate the model.

**Figure 11:**
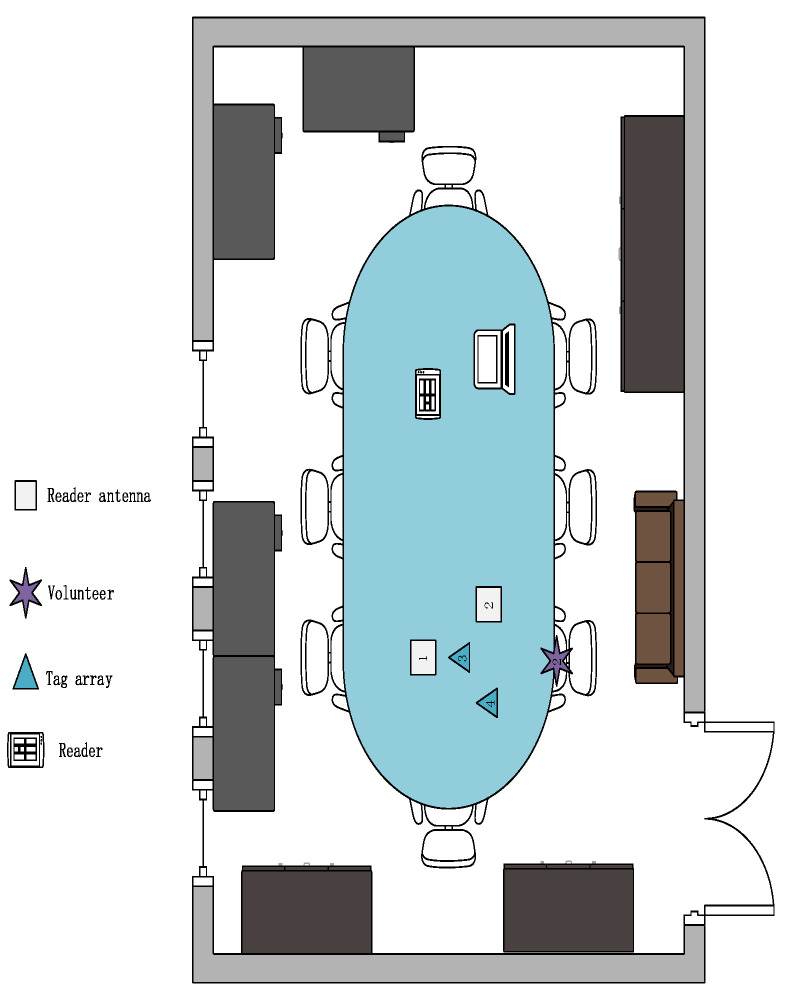
A picture of the lab scene in a conference room.

**Figure 12:**
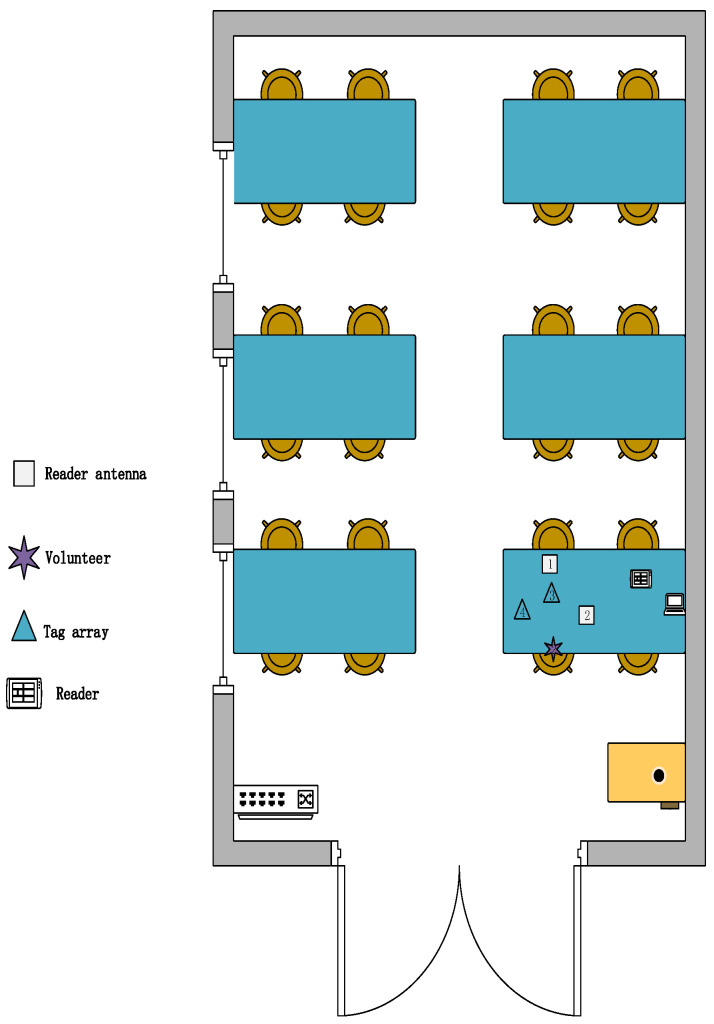
Classroom experiment scene diagram.

**Figure 13:**
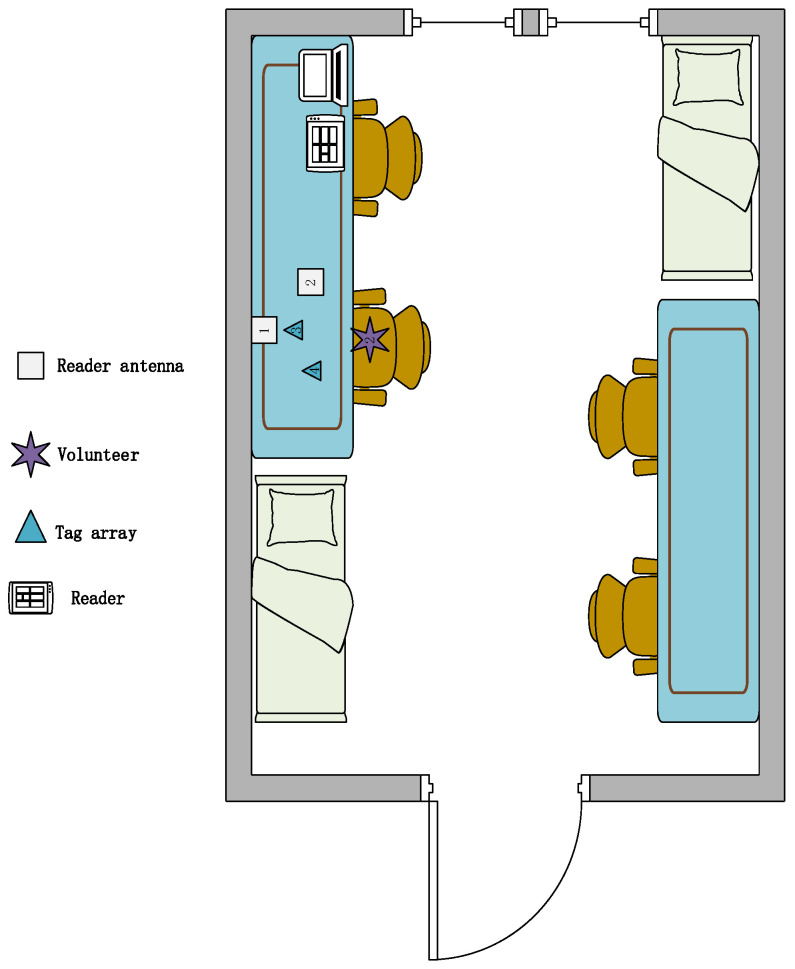
Dormitory experiment scene map.

**Environment:** We designed three experimental scenarios: for scenario A, as shown in Figure 11, the user drew gestures in a conference room with a length of 8.5 m and a width of 7.2 m. Only a small amount of electronic devices and metal interference existed in the conference room. For scenario B, as shown in Figure 12, the user conducts experiments in a classroom with a length of 10 m and a width of 7 m, where there are several groups of tables and chairs, and the material of the tables and chairs is mainly metal and wood, so there is a lot of metal interference in this experimental scene. For scenario C, as shown in Figure 13, the user conducts gesture drawing in a dormitory with a length of 8.5 m and a width of 4.5 m, and there are many electronic devices and metal brackets in the dormitory.

### 5.1. Accuracy of Different English Letters

**Figure 14:**
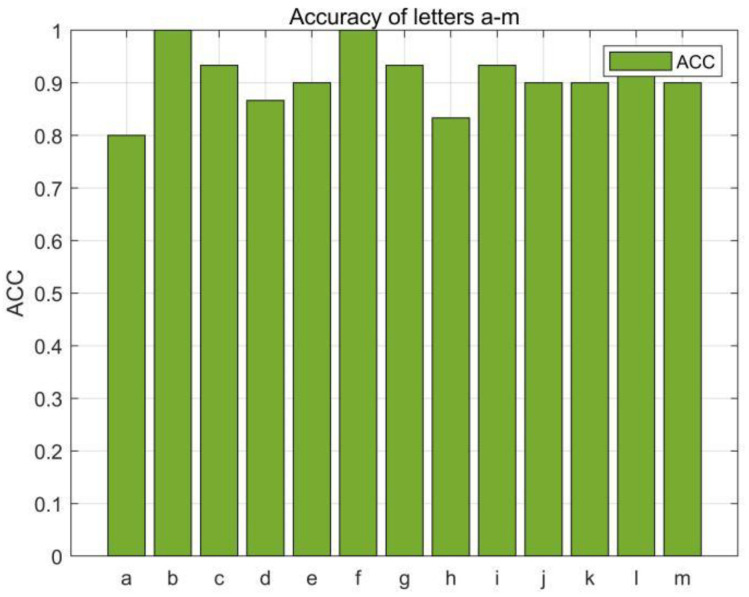
Accuracy of the letters “a–m”.

**Figure 15:**
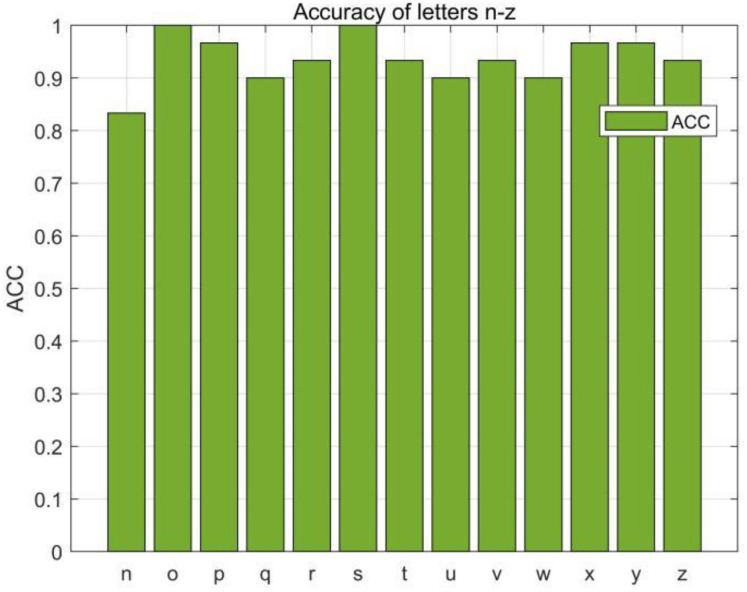
Accuracy of the letters “n–z”.

In this section, we first evaluated the accuracy of each English letter, we selected 780 data (30 copies of each English letter) from the collected dataset for the RF-alphabet, and the experimental results are shown in Figure 14 and Figure 15, where two groups of letters, a and d, n and h, are more similar due to the front and side drawing gestures of the letters within each group, and the collected phase of the difference between the waveforms is not great, and the difference only lies in the size of the phase values of some feature points, so the accuracy is low at 83.33%, but the average accuracy of the other letters reaches 94.08%, and the overall average accuracy is 92.43%.

### 5.2. Accuracy under Different Users

**Figure 16:**
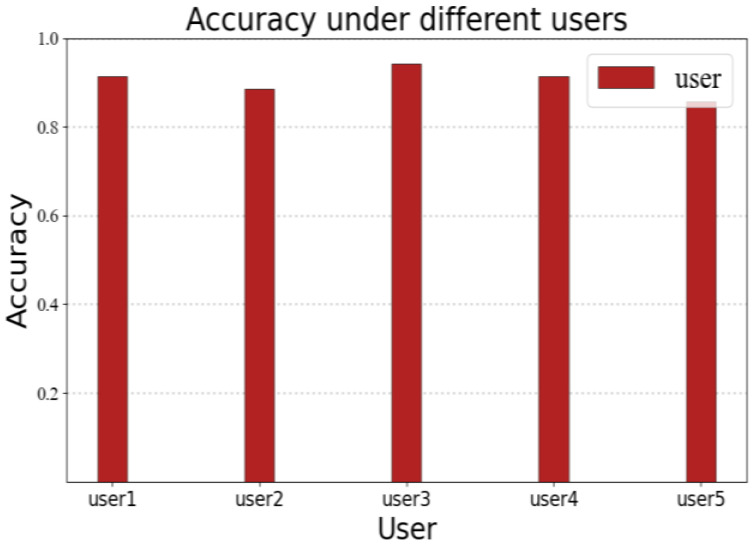
Accuracy rate under different users.

In order to verify the accuracy of RF-alphabet under different users, we invited five volunteers to draw A–Z in scene A, respectively, and each volunteer drew a total of 35 times (the number of times each volunteer drew each letter in 26 letters was different); the experimental results are shown in Figure 16, among which the accuracy of volunteers 1, 3, and 4 were 91.42%, 94.28% and 91.42%. The accuracy rates of volunteers 2 and 5 were 88.57% and 85.71%, respectively, due to the presence of multiple confused letters among the 30 letters drawn by volunteers 2 and 5 (where user 2 drew three times a, two times d, and two times n; volunteer 5 drew four times a, three times h, and one time n), but the system still provided an average accuracy rate of 90.28%.

### 5.3. Accuracy under Different Environments

**Figure 17:**
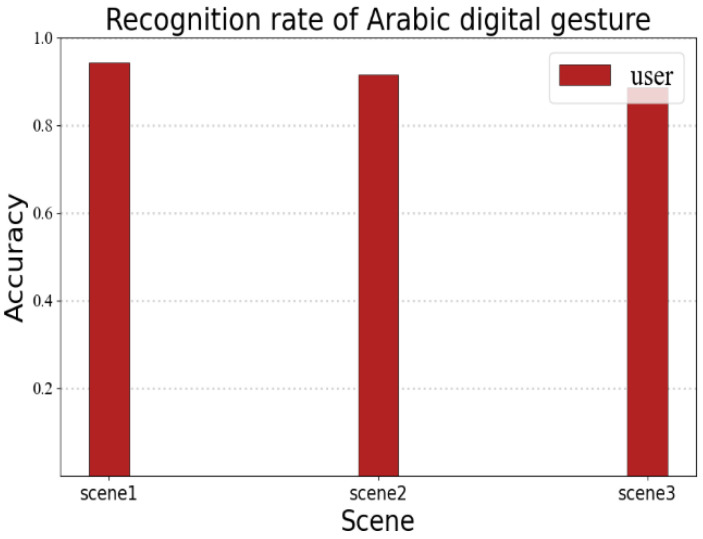
Accuracy in different environments.

To verify the accuracy of the RF-alphabet in different environments, we invited one volunteer to draw the letters A–Z in three environments, A, B, and C. To control the variables, we asked the volunteer to draw each letter twice, for a total of 52 drawings in each environment. Figure 17 shows the accuracy of the letters drawn by this volunteer in the three different environments, with the highest accuracy of 92.3% in scene A, followed by scene B with 90.38% accuracy, and the lowest accuracy of 86.53% in scene C. This is due to the presence of a large amount of metal and electronic device interference in scene C, which interferes with the collected phase, especially for confusing letters, but the system can still provide an average accuracy of 89.7%.

### 5.4. RF-Alphabet Recognition of Arabic Numerals

**Figure 18:**
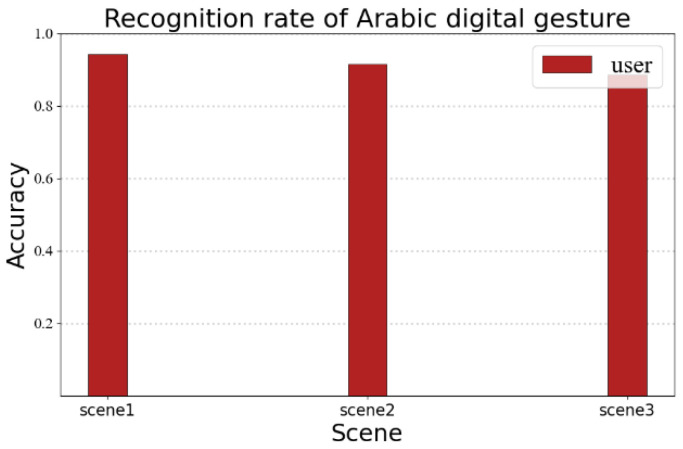
Recognition rate of Arabic digital gesture.

The recognition performance may vary for different linguistic information. In order to verify the portability of the RF-alphabet, we conducted a validation experiment for Arabic numerals. We invited a volunteer to draw Arabic numerals 0–9, and the volunteer drew each number 35 times in three different experimental environments, as shown in Figure 18; the average recognition rates of users’ gestures in the three environments were 94.2%, 91.4%, and 88.5%, respectively. It can be seen that RF-alphabet can effectively recognize not only English letters, but also Arabic numerals.

### 5.5. Comparison with the Latest Methods

Finally, we compared the latest gesture recognition algorithms, the comparison results are shown in Table 2. The RF-alphabet has higher accuracy than RF-FreeGR [29] and FingerPass in different environments and with different users, and RF-alphabet uses fewer tags to achieve better accuracy compared to RF-FreeGR, although FingerPass [25] uses WIFI instead of RFID, thus reducing hardware dependency, the model uses a deep learning-based algorithm, and FingerPass requires more training data and is less efficient compared to the model-based RF-alphabet.

## 6. Discussion

The RF-alphabet quickly classifies and recognizes alphabetic gesture information by the Dtsc model. However, the RF-alphabet has some limitations in practical applications. First, the RF-alphabet achieves gesture recognition of single letters, but not of consecutive English words. The difficulty of gesture recognition for consecutive English words is the segmentation and detection when transitioning between different letters. In the future, we intend to use data segmentation and threshold detection techniques to split words into individual letters and splice the split letters to achieve gesture recognition of consecutive words.

Secondly, the RF-alphabet requires more gesture data for different users to accurately recognize the drawn letters. In the future, we intend to use reinforcement learning to achieve maximum gesture recognition of letters during interaction with different users. We also consider using GAN adversarial neural networks to retain information related to specific letter gestures during the adversarial learning process to improve the generalization ability of the model. The effect of data expansion and cross-domain recognition is achieved to accurately recognize the user’s gestures.

## 7. Summary

The RF-alphabet proposed in this paper is an RFID-based, device-free, domain-independent gesture recognition system for the alphabet. It is capable of recognizing all letters in the alphabet. Our proposed dual-antenna, the dual-tag layout is able to capture alphabet gestures in all directions, overcoming the limitations of traditional tag arrays. By de-noising and analyzing the captured RFID gesture data, data normalization and data smoothing are performed. Moreover, this paper designs the Dtsc model to classify and identify the pre-processed data by combining the differential thresholding method and similarity calculation. After extensive experiments, it is shown that the RF-alphabet is able to recognize complex, fine-grained, domain-independent alphabetic gestures. The overall accuracy rates are 90.28% and 89.7% for different users and sites with different environments. The system we designed is significantly due to existing solutions, and we believe that the RF-alphabet can facilitate RFID-based gesture recognition for human-computer interaction.

## Figures and Tables

**Table 1:** Experimental parameters table.

Parameters	Value
Spacing between tag and antenna	30 cm
The angle between the antenna and the ground	90 °C
Frequency of the reader	920.875 MHz

**Table 2:** Comparison with the latest methods.

	RF-FreeGR	FingerPass	RF-Alphabet
Accuracy in different environments	88.38%	87.6%	89.7%
Accuracy under different users	90.02%	89.8%	90.28%
Number of labels	4 × 3 Label Array	/	2 Tags
Signals	RFID	WiFi	RFID
System Design Methodology	Based on deep learning algorithms	Based on deep learning algorithms	Model-based design algorithms

## Data Availability

Not applicable.
